# ﻿Two new *Cordyceps*-like species, *Perennicordycepszongqii* sp. nov. (Polycephalomycetaceae) and *Purpureocilliumzongqii* sp. nov. (Ophiocordycipitaceae), in Hypocreales from karst region of China

**DOI:** 10.3897/mycokeys.110.135724

**Published:** 2024-11-07

**Authors:** Wan-Hao Chen, Dan Li, Jian-Dong Liang, Xiu-Xiu Ren, Jie-Hong Zhao, Yan-Feng Han

**Affiliations:** 1 Center for Mycomedicine Research, Basic Medical School, Guizhou University of Traditional Chinese Medicine, Guiyang 550025, Guizhou, China; 2 Institute of Fungus Resources, Department of Ecology, College of Life Sciences, Guizhou University, Guiyang 550025, Guizhou, China; 3 Key Laboratory of Microbio and Infectious Disease Prevention & Control in Guizhou Province, Guiyang 550025, Guizhou, China; 4 College of Pharmacy, Guizhou University of Traditional Chinese Medicine, Guiyang 550025, Guizhou, China

**Keywords:** *Cordyceps*-like species, morphology, Ophiocordycipitaceae, phylogenetic analysis, Polycephalomycetaceae

## Abstract

Two new *Cordyceps*-like species, *Perennicordycepszongqii* and *Purpureocilliumzongqii*, isolated from a larva and soil, are introduced. Morphological comparisons and phylogenetic analyses based on multigene datasets (ITS, LSU, *RPB2* and *TEF*) support the establishment of the new species. Moreover, new species in the families Polycephalomycetaceae and Ophiocordycipitaceae were introduced into Tiankeng and the valley for the first time. Further attention needs to be paid to the diversity of other *Cordyceps*-like fungi in the special eco-environment of the karst region.

## ﻿Introduction

*Cordyceps*-like fungi, also known as *Cordyceps* sensu lato, refers to species belonging to Hypocreales, Sordariomycetes and Ascomycota, and contains all the species in the families Cordycipitaceae, Ophiocordycipitaceae and Polycephalomycetaceae, as well as some species in the family Clavicipitaceae (Li et al. 2021; Xiao et al. 2023). Currently, more than 2000 species of *Cordyceps*-like fungi have been reported worldwide, while there are just over 300 species in China (Chen et al. 2021d; Li et al. 2023, http://www.indexfungorum.org/Names/Names.asp, 17 August, 2024). Therefore, more attention needs to be paid to the diversity of *Cordyceps*-like fungi in China.

The karst region, especially in southern China, preserves unique, large-scale, and continuously distributed primitive forests with extremely rich biodiversity. The complex ecological environment and special geographical conditions in the region have become shelters for many unique species (Özkan et al. 2010; Su et al. 2017). Previous studies have shown that the resources of *Cordyceps*-like fungi in karst areas are very abundant (Zhu et al. 2004; Song et al. 2011). In recent years, Yunnan and Guizhou Province have become hot areas for research on *Cordyceps*-like fungi (Ming et al. 2021; Chen et al. 2021a, b, c, 2022a, b, c, 2023; Qu et al. 2021; Dong et al. 2022; Wang et al. 2022, 2024a; Zhou et al. 2022; Zou et al. 2022; Peng et al. 2023, 2024; Tang et al. 2023a, b, c; Xiao et al. 2023, 2024; Zhang et al. 2023; Dai et al. 2024), and the majority of sampling locations were located in karst forest habitats. However, there are also some special habitats in karst areas, such as Tiankeng and the valley, because of its unique geological landscape, which creates a microclimate distinct from its surrounding area and a unique habitat suitable for biological survival. Unfortunately, the *Cordyceps*-like fungi in these habitats have been neglected.

Chen et al. (2022b, 2023) reported ten new *Cordyceps*-like fungi in the family Cordycipitaceae from Tiankeng and the valley. Two new genera and three new species in the family Clavicipitaceae were introduced from the valley by Chen et al. (2022a). However, *Cordyceps*-like fungi were rarely reported in other families from Tiankeng or the valley. Besides, *Cordyceps*-like fungi was omnipresent in the obtaining nutrients and was abundant in the soil (Chen et al. 2022a, Quesada-Moraga et al. 2007). Unfortunately, few reports exist about the *Cordyceps*-like fungi from the soil of Tiankeng or the valley.

During a survey of *Cordyceps*-like fungi associated with insects and soil from Southwest China, the infected specimen and soil were collected, and strains were isolated. After detailed multiloci phylogenic analysis and morphological observations, two new species were identified as belonging to the family Polycephalomycetaceae and Ophiocordycipitaceae.

## ﻿Materials and methods

### ﻿Specimen and soil collection, isolation

The specimen and soil (for the photo descriptions of the sampling site see Suppl. materials 1–5) were collected from Mayao River Valley (26°22'8.3748"N, 107°23'16.96"E), Duyun City, Qiannan Buyei and Miao Autonomous Prefecture and Monkey-Ear Tiankeng (27°5'12.138"N, 107°0'48.42"E), Kaiyang County, Guiyang, Guizhou Province, on 1 May 2022 and 19 July 2023. The samples were placed in an ice box and brought to the laboratory. Specimens were preserved in the refrigerator at 4 °C until further processing. The surface of each arthropod body was rinsed with sterile water, followed by sterilization with 75% ethanol for 3–5 s and rinsing again three times with sterilized water. After drying on sterilized filter paper, a piece of the synnemata, mycelium or sclerotia was cut from the specimen and inoculated on agar plates of potato dextrose agar (PDA) or PDA modified by the addition of 1% w/v peptone containing 0.1 g/l streptomycin and 0.05 g/l tetracycline (Chen et al. 2019). After fungal colonies emerged from the inoculated samples, a piece of mycelium from the colony edge was transferred onto new agar plated and cultured at 25 °C for 14 days under 12 h light/12 h dark conditions (Zou et al. 2010). Then 2 g collected soil were placed into a sterile conical flask containing 20 ml sterile water and thoroughly shaken using a Vortex vibration meter. Next, the suspension was diluted to a concentration of 10^-3^. Subsequently, 1 ml of the diluted sample was added to a sterile Petri dish and mixed with Sabouraud’s dextrose agar (SDA; peptone 10 g/l, dextrose 40 g/l, agar 20 g/l, 3.3 ml of 1% Bengal red aqueous solution) medium containing 50 mg/l penicillin and 50 mg/l streptomycin. After the plates were incubated at 25 °C for 1–2 weeks, single colonies were transferred from the plates to new PDA plates (Wang et al. 2024b). The holotypes and ex-types were deposited at the Institute of Fungus Resources, Guizhou University (formally Herbarium of Guizhou Agricultural College; code, GZAC), Guiyang City, Guizhou, China. MycoBank numbers have been obtained as outlined in MycoBank (http://www.MycoBank.org) (Crous et al. 2004).

### ﻿Morphological studies

Colony morphology was determined on PDA cultures incubated at 25 °C for 14 days and the growth rate, the presence of octahedral crystals and the colony colours (surface and reverse) were observed. To investigate the microscopic characteristics, a little of the mycelia was picked up from the colony and mounted in lactophenol cotton blue or 20% lactate acid solution and the asexual morphological characteristics (e.g., conidiophores, phialides and conidia) were observed and measured using a Leica DM4 B microscope. Twenty measurements were recorded for hyphae, conidiophores, phialides and conidium.

### ﻿DNA extraction, PCR and sequencing

DNA extraction was carried out using a fungal genomic DNA extraction kit (DP2033, BioTeke Corporation) according to Liang et al. (2011). The extracted DNA was stored at −20 °C. Polymerase chain reaction (PCR) was used to amplify genetic markers using the following primer pairs: ITS4/ITS5 for the internal transcribed spacer (ITS) region (White et al. 1990), LR0R/LR5 for 28s large subunit ribosomal (LSU) (Vilgalys and Hester 1990), fRPB2-5F/fRPB2-7cR for RNA polymerase II second largest subunit (*RPB2*) (Liu et al. 1999) and 983F/2218R for translation elongation factor 1 alpha (*TEF*) (Castlebury et al. 2004). The thermal cycle of PCR amplification for these phylogenetic markers was set up following the procedure described by Chen et al. (2021c). PCR products were purified and sequenced at Sangon Biotech (Shanghai) Co. by Sanger dideoxy sequencing. All newly generated sequences were deposited in GenBank and accession numbers were obtained. The sequences used in the study were listed in Table 1.

**Table 1.** List of strains and GenBank accession numbers of sequences used in this study.

Species	Strain	Host/ substrate	GenBank Accession Number				Reference
			ITS	LSU	RPB2	TEF	
*Perennicordycepscuboidea*	NBRC 103836	Larva of beetle	JN943332	JN941420	AB972955	AB972951	Schoch et al. 2012
*Perennicordycepscuboidea*	NBRC 100941	stroma of *Cordycepsstylophora*	JN943329	JN941416	–	–	Schoch et al. 2012
*Perennicordycepscuboidea*	NBRC 101740	Larva of beetle	JN943331	JN941417	–	KF049684	Schoch et al. 2012
*Perennicordycepselaphomyceticola*	MFLU 21-0264	*Elaphomyces* sp.	OQ172067	OQ172035	OQ459794	OQ459720	Xiao et al. 2023
*Perennicordycepselaphomyceticola*	MFLU 21-0263	*Elaphomyces* sp.	OQ172065	OQ172033	OQ459793	OQ459719	Xiao et al. 2023
*Perennicordycepselaphomyceticola*	MFLU 21-0262	*Elaphomyces* sp.	OQ172064	OQ172032	OQ459792	OQ459718	Xiao et al. 2023
*Perennicordycepslutea*	KUMCC 3004^T^	*Ophiocordycepssinensis*	–	OQ474910	–	–	Xiao et al. 2023
*Perennicordycepsparacuboidea*	NBRC 101742^T^	Larva of beetle	JN943338	JN941431	KF049669	KF049685	Ban et al. 2015a
*Perennicordycepsparacuboidea*	NBRC 100942	Larva of beetle	JN943337	JN941430	AB972958	AB972954	Ban et al. 2015a
*Perennicordycepsprolifica*	NBRC 101750	Larva of *Tannajaponensis*	JN943340	JN941433	AB972957	AB972953	Ban et al. 2009
*Perennicordycepsprolifica*	NBRC 100744	Larva of *Tannajaponensis*	AB925942	JN941432	AB972956	AB972952	Ban et al. 2009
*Perennicordycepsprolifica*	TNS-F-18547	Larvae of cicada	KF049660	KF049632	KF049670	KF049687	Kepler et al. 2013
*Perennicordycepsryogamiensis*	NBRC 101751	Larva of beetle	JN943343	JN941438	–	KF049688	Schoch et al. 2012
*Perennicordycepsryogamiensis*	NBRC 103837	Larva of beetle	JN943346	JN941439	–	–	Schoch et al. 2012
*Perennicordycepsryogamiensis*	NBRC 103842	*Cordycepsryogamiensis*	JN943345	JN941440	–	–	Schoch et al. 2012
***Perennicordycepszongqii***	**DY05421^T^**	**Larva of moth**	**PQ211278**	**PQ211282**	**PQ223677**	**PQ223679**	This study
***Perennicordycepszongqii***	**DY05422**	**Larva of moth**	**PQ211279**	**PQ211283**	**PQ223678**	**PQ223680**	This study
*Pleurocordycepsparvicapitata*	MFLU 21-0270	*Elaphomyces* sp.	OQ172082	OQ172054	OQ459796	OQ459722	Xiao et al. 2019
*Pleurocordycepsparvicapitata*	MFLU 21-0271^T^	*Elaphomyces* sp.	OQ172083	OQ172055	OQ459797	OQ459723	Xiao et al. 2023
*Pleurocordycepssinensis*	HMAS 43720^T^	Larvae of *Hepialusarmocanus*	NR_119928	NG_042573	–	–	Sun et al. 2019
*Pleurocordycepsvitellina*	KUMCC 3006^T^	*Ophiocordyceps* *nigrella*	OQ172089	OQ172061	OQ459803	OQ459729	Xiao et al. 2023
*Pleurocordycepsvitellina*	KUMCC 3007	*Ophiocordyceps* *nigrella*	OQ172090	OQ172062	OQ459804	OQ459730	Xiao et al. 2023
*Polycephalomycesformosus*	NBRC 109993^T^	Larvae of Coleoptera	MN586833	MN586842	MN598064	MN598057	Wang et al. 2021
*Polycephalomycesalbiramus*	GACP 21-XS08^T^	Gryllotalpa	OQ172092	OQ172037	OQ459807	OQ459735	Xiao et al. 2023
*Polycephalomycesalbiramus*	GACPCC 21-XS08	Gryllotalpa	OQ172093	OQ172038	OQ459808	OQ459736	Xiao et al. 2023
*Purpureocilliumatypicola*	CBS 744.73	*Atypuskarschi*	GU980041	EF468841	–	EF468786	Perdomo et al. 2013
*Purpureocilliumjiangxiense*	JX17D04	Soil	PP555636	PP555645	–	PP658209	Chen et al. 2024
*Purpureocilliumjiangxiense*	JX13B01^T^	Soil	PP555637	PP555646	–	PP658210	Chen et al. 2024
*Purpureocilliumlavendulum*	FMR 10376^T^	Soil	FR734106	–	–	FR775516	Perdomo et al. 2013
*Purpureocilliumlavendulum*	CBS 128678	Human	MH864977	MH876430	–	–	Perdomo et al. 2013
*Purpureocilliumlilacinum*	CBS 284.36^T^	Soil	FR734101	–	–	FR734156	Perdomo et al. 2013
*Purpureocilliumlilacinum*	FMR 8652	Human	FR734090	FR775473	–	–	Perdomo et al. 2013
*Purpureocilliumroseum*	IOM 325363.1	Human	MT560195	MT560197	–	–	Calvillo‐Medina et al. 2021
*Purpureocilliumroseum*	IOM 325363.2	Human	MT560196	MT560198	–	–	Calvillo‐Medina et al. 2021
*Purpureocilliumsodanum*	IBRC-M 30175^T^	Salt crystals	KX668542	–	–	–	Hyde et al. 2016
*Purpureocilliumtakamizusanense*	NBRC 100742	*Tannajaponensis*	LC008197	–	–	LC008333	Ban et al. 2015b
*Purpureocilliumtakamizusanense*	NBRC 108982	Cicada	LC008204	–	–	LC008338	Ban et al. 2015b
*Purpureocilliumtakamizusanense*	NBRC 110232	-	LC008205	–	–	LC008339	Ban et al. 2015b
***Purpureocilliumzongqii***	**TK041^T^**	**Soil**	**PQ211280**	**PQ211284**	–	**PQ223681**	This study
***Purpureocilliumzongqii***	**TK042**	**Soil**	**PQ211281**	**PQ211285**	–	**PQ223682**	This study
*Simplicilliumlamellicola*	CBS 116.25^T^	*Agaricusbisporus*	MH854806	AF339552	DQ522462	DQ522356	Luangsa-ard et al. 2011
*Simplicilliumlanosoniveum*	CBS 704.86	*Hemileiavastatrix*	AJ292396	AF339553	DQ522464	DQ522358	Luangsa-ard et al. 2011

Note: New strains or species are in bold type.“^T^” denotes ex-type. Abbreviations for collections: CBS, Centraalbureau voor Schimmelcultures, Utrecht, the Netherlands; TNS-F, the mycological herbarium of the National Museum of Nature and Science, Tsukuba, Ibaraki, Japan; HMAS, Herbarium of Mycology, Chinese Academy of Sciences; IBRC, Iranian Biological Resource Center, Tehran, Iran; NBRC, NITE Biological Resource Center, Sayaka Ban National Institute of Technology and Evaluation, Japan; IOM, Instituto de Oftalmología ‘Fundación Conde de Valenciana’ IAP Mexico Culture Collection; MFLU, Mae Fah Luang University; GACP, Herbarium of Guizhou University, China; KUMCC, Culture collection of Kunming Institute of Botany, Kunming, China; FMR, Facultad de Medicina, Reus, Tarragona, Spain.

### ﻿Sequence alignments and phylogenetic analyses

DNASTAR™ Lasergene (v 6.0) was used to edit DNA sequences in this study. The ITS, LSU, *RPB2* and *TEF* sequences were downloaded from GenBank, based on Xiao et al. (2023), Calvillo‐Medina et al. (2021), Luangsa-ard et al. (2011), Chen et al. (2024) and others selected based on BLASTn searches in GenBank. ITS sequences and other loci were aligned and edited by MAFFT v.7.037b (Katoh and Standley 2013) and MEGA6 (Tamura et al. 2013). Combined sequences of ITS, LSU, *RPB2* and *TEF* were obtained using SequenceMatrix v.1.7.8 (Vaidya et al. 2011). The model was selected for Bayesian analysis by ModelFinder (Kalyaanamoorthy et al. 2017) in PhyloSuite (v1.2.2) software (Zhang et al. 2020).

The combined dataset of ITS, LSU, *RPB2* and *TEF* sequence data (Suppl. materials 1–5) was analyzed using Bayesian inference (BI) and maximum likelihood (ML) methods. For BI, a Markov chain Monte Carlo (MCMC) algorithm was used to generate phylogenetic trees with Bayesian probabilities for the combined sequence datasets using MrBayes v.3.2 (Ronquist et al. 2012). The Bayesian analysis resulted in 20,001 trees after 10,000,000 generations. The first 4,000 trees, representing the burn-in phase of the analysis, were discarded, while the remaining 16,001 trees were used to calculate posterior probabilities in the majority rule consensus tree. After the analysis was finished, each run was examined if it was greater than 200 using the program Tracer v.1.5 (Drummond and Rambaut 2007) to determine burn-in and confirm that both runs had converged. ML analyses were constructed with IQ-TREE (v 2.0) (Trifinopoulos et al. 2016), using an automatic selection of the model according to BIC.

## ﻿Results

### ﻿Phylogenetic analyses

**Figure 1.**
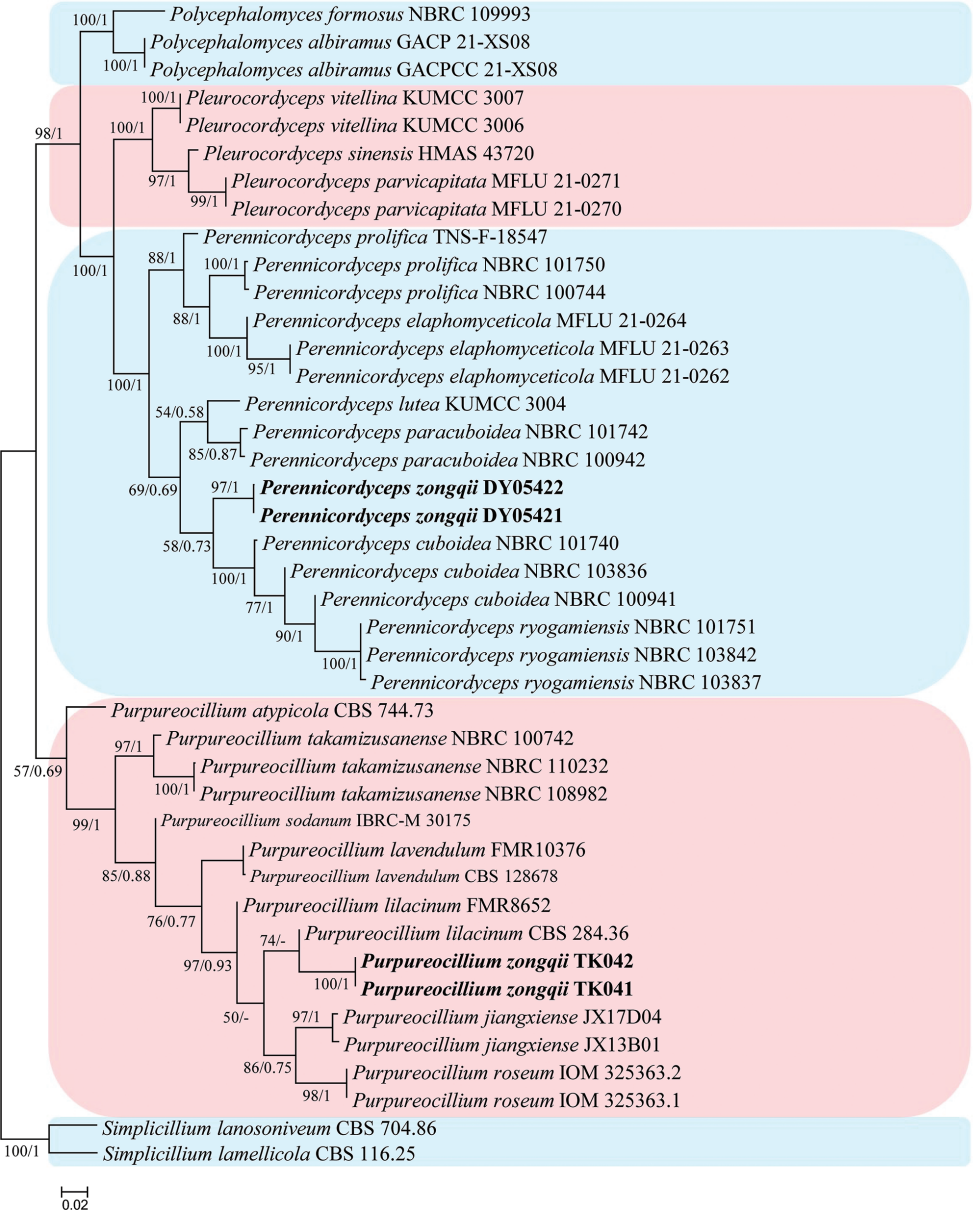
Phylogenetic analysis of the new strains and its related species based on multigene dataset (ITS, LSU, *RPB2* and *TEF*). Statistical support values (≥ 50%/0.5) are shown at the nodes for ML bootstrap support/BI posterior probabilities.

The phylogenetic tree (Fig. 1) was generated to determine the relationship among those new strains and its related species. *Simplicilliumlanosoniveum* (J.F.H. Beyma) Zare & W. Gams (CBS 704.86) and *S.lamellicola* (F.E.V. Sm.) Zare & W. Gams (CBS 116.25) were used as the outgroups. The concatenated sequences included 40 taxa and consisted of 3,392 (ITS, 616; LSU, 841; *RPB2*, 1,041; and *TEF*, 894) characters with gaps.

The selected model for ML analysis was TN+F+I+G4. The final value of the highest scoring tree was –15,988.941, which was obtained from an ML analysis of the dataset (ITS, LSU, *RPB2* and *TEF*). The parameters of the rate heterogeneity model used to analyze the dataset were estimated using the following frequencies: A = 0.230, C = 0.288, G = 0.278, T = 0.204; substitution rates AC = 1.00000, AG = 2.57904, AT = 1.00000, CG = 1.00000, CT = 5.70221 and GT = 1.00000, as well as the gamma distribution shape parameter α = 0.805. The selected models for BI analysis were GTR+F+I+G4 (ITS, LSU, *RPB2* and *TEF*). The phylogenetic trees (Fig. 1) constructed using ML and BI analyses were largely congruent and strongly supported in most branches. The new strains DY05421 and DY05422 were clustered into an independent clade in the group of the genus *Perennicordyceps* and have a close relationship with *Perennicordycepscuboidea* (Kobayasi & Shimizu) Matočec & I. Kušan (NBRC 101740, NBRC 103836 and NBRC 100941) and *P.ryogamiensis* (Kobayasi & Shimizu) Matočec & I. Kušan (NBRC 101751, NBRC 103842 and NBRC 103837). Strains TK041, TK042 were clustered into the group of the genus *Purpureocillium* and have a close relationship with *Purpureocilliumlilacinum* (Thom) Luangsa-ard, Houbraken, Hywel-Jones & Samson (CBS 284.36).

### ﻿Taxonomy

#### ﻿ Perennicordyceps zongqii

Taxon classificationFungiHypocrealesOphiocordycipitaceae

W.H. Chen, Y.F. Han & J.D. Liang sp. nov.

237B91C0-8B65-5F56-A847-B9BFD5874389

855564

Fig. 2

##### Etymology.

In honor of Prof. Zongqi Liang, for his support and guidance in arthropod pathogenic fungi research.

**Figure 2.**
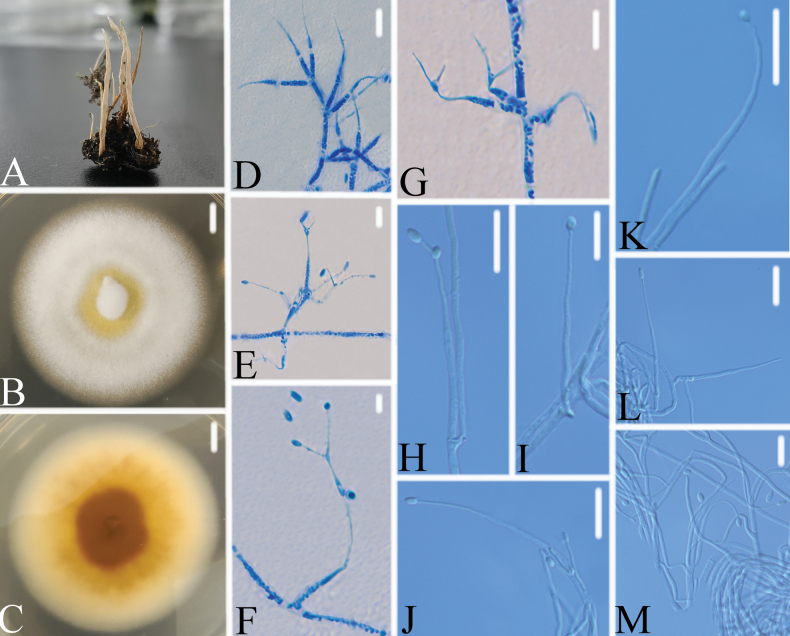
*Perennicordycepszongqii***A** infected larva **B**, **C** colony on PDA (**B** obverse, **C** reverse) **D–M** phialides and conidia. Scale bars: 10 mm (**B**, **C**); 10 μm (**D–M**).

##### Type.

China • Guizhou Province, Qiannan Buyei and Miao Autonomous Prefecture, Duyun City, Mayao River Valley (26°22'8.3748"N, 107°23'16.96"E), on a larva of moth (Lepidoptera), on the leaf litter, 1 May 2022, Wanhao Chen, GZAC DY0542 (holotype), ex-type DY05421.

##### Description.

Colonies on PDA, attaining a diameter of 56–59 mm after 14 days at 25 °C, white, consisting of a basal felt, floccose hyphal overgrowth, yellowish in middle; reverse yellow to pale yellowish, light brown to brown in the middle. Hyphae septate, hyaline, yellowish in the middle part of the colony, smooth-walled, 1.1–2.1 μm wide. Conidiophores erect, hyaline, irregular branched, with 1–4 phialides. Phialides 29.3–31.1 × 1.5–2.4 μm, hyaline, cylindrical at base, gradually tapering near the apex, holoblastic or branch. Conidia 3.4–4.8 × 2.5–2.7 μm, hyaline, smooth-walled, thin-walled, ellipsoidal to cylindrical, unicellular, acuminate, arranged in chains not observed.

##### Distribution.

Duyun City, Guizhou Province, China.

##### Host.

Larva (Lepidoptera).

##### Additional strain examined.

China • Guizhou Province, Qiannan Buyei and Miao Autonomous Prefecture, Duyun City, Mayao River Valley (26°22'8.3748"N, 107°23'16.96"E). On a larva of moth (Lepidoptera), on the leaf litter, 1 May 2022, Wanhao Chen, DY05422 (living culture).

##### Notes.

Strain DY05421 was easily identified as *Perennicordyceps*, based on the BLASTn result in NCBI. Phylogenetic analyses show that strain DY05421 has close relationships to *P.cuboidea and P.ryogamiensis* (Fig. 1). However, strain DY05421 was easily distinguished from *P.cuboidea* (globose to ellipsoid conidia, 1.3–3.7 × 1.1–2.3 μm; phialide, 18.9–22.2 × 0.8–1.1 μm; substrate, larvae of Coleoptera) by its larger ellipsoidal to cylindrical conidia, larger phialide and the substrate (Ban et al. 2009). Strain DY05421 was easily distinguished from *P.ryogamiensis* (ellipsoid conidia, 2.5–3.9 × 1.0–1.4 μm; phialide, 17.5–55.2 × 0.8–2.7 μm; substrate, larvae of Coleoptera) by its larger ellipsoidal to cylindrical conidia, smaller phialide and the substrate (Ban et al. 2009). Thus, the morphological characteristics and molecular phylogenetic results support *strain* DY05421 as a new *Perennicordyceps* species and named *Perennicordycepszongqii*.

#### ﻿ Purpureocillium zongqii

Taxon classificationFungiHypocrealesOphiocordycipitaceae

W.H. Chen, Y.F. Han & J.D. Liang sp. nov.

0DA9780F-77E4-5B0C-836D-FF09EFFD1C7C

855565

Fig. 3

##### Etymology.

In honor of Prof. Zongqi Liang, for his support and guidance in arthropod pathogenic fungi research.

**Figure 3.**
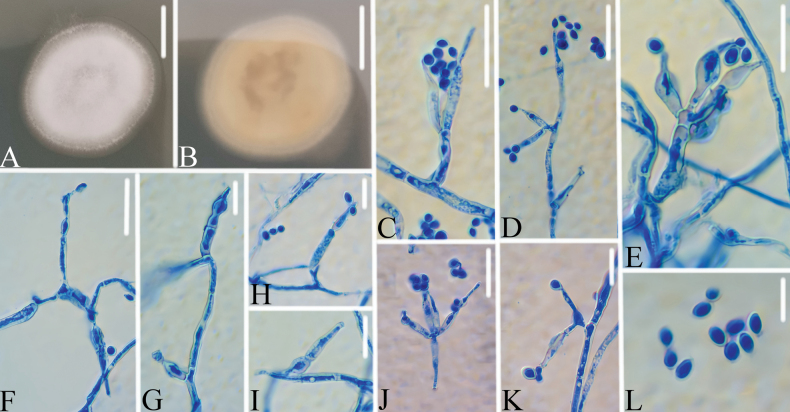
*Purpureocilliumzongqii***A**, **B** colony on PDA (**A** obverse, **B** reverse) **C–J** Phialides and conidia. Scale bars: 10 mm (**A**, **B**); 10 μm (**C–J**).

##### Type.

China • Guizhou Province, Guiyang, Kaiyang County, Monkey-Ear Tiankeng (27°5'12.138"N, 107°0'48.42"E), soil, 19 July 2023, Wanhao Chen, GZAC TK04 (dried holotype), ex-type TK041.

##### Description.

Colonies on PDA, attaining a diameter of 23–25 mm after 14 days at 25 °C, white, consisting of a basal felt, floccose hyphal overgrowth, white; reverse yellowish. Hyphae septate, hyaline, smooth-walled, 1.1–1.9 μm wide. Conidiophores 9.3–12.7 × 2.1–2.4 μm, erect, hyaline, verticillately branched, with 1–4 phialides. Phialides 6.8–11.7 × 2.6–4.0 μm, hyaline, cylindrical at base, gradually tapering near the apex. Conidia 2.7–4.2 × 2.0–2.4 μm, hyaline, smooth-walled, thin-walled, ellipsoidal, unicellular, acuminate, arranged in chains not observed.

##### Substrate.

Soil.

##### Distribution.

Kaiyang County, Guizhou Province, China.

##### Additional strain examined.

China • Guizhou Province, Guiyang, Kaiyang County, Monkey-Ear Tiankeng (27°5'12.138"N, 107°0'48.42"E), soil, 19 July 2023, Wanhao Chen, TK042 (living culture).

##### Notes.

*Purpureocilliumzongqii* was easily identified as *Purpureocillium*, based on the BLASTn result in NCBI. Phylogenetic analyses show that *P.zongqii* has a close relationship to *P.lilacinum* (Fig. 1). However, *P.zongqii* was easily distinguished from *P.lilacinum* (conidiophores: 4–6 × 3–4 μm; conidia: ellipsoidal to fusiform, 2–3 × 2–4 μm; phialide, 6–9 × 2.5–3 μm; purple colony) (Luangsa-ard et al. 2011) by its larger conidiophores, larger ellipsoidal conidia, larger phialide and white colony. Thus, the morphological characteristics and molecular phylogenetic results support *P.zongqii* as a new species.

### ﻿Keys of the genus *Perennicordyceps*

**Table:** 

1	Parasitic on fungi	**2**
–	Parasitic on insects	**3**
2	Phialides 14.8–64.9 × 1.9–3.1 μm, conidia globose to ellipsoid	***Perennicordycepslutea***
–	Phialides 12–16 × 0.6–1.5 μm, conidia fusiform to ellipsoid to inequilateral shaped	***Perennicordycepselaphomyceticola***
3	Typical host larva of cicada or moth	**4**
–	Typical host larva of beetle	**5**
4	Typical host larva of cicada, conidia globose, fusiform 1.5–3.5 (2.5) × 1.1–1.8 (1.4) μm	***Perennicordycepsprolifica***
–	Typical host larva of moth, conidia ellipsoidal to cylindrical, 3.4–4.8 × 2.5–2.7 μm	***Perennicordycepszongqii***
5	Conidiophores quasi-verticillate branching	**6**
–	Conidiophores irregular branching	***Perennicordycepscuboidea***
6	Conidia ellipsoid, 2.5–3.9 (3.1) × 1.0–1.4 (1.2) μm, mainly *Acremonium*-like phialide	***Perennicordycepsryogamiensis***
–	Conidia aubglobose, fusiform, 1.3–1.9 (1.8) × 1.0–1.9 (1.4) μm, mainly *Hirsutella*-like phialide	***Perennicordycepsparacuboidea***

### ﻿Keys of the genus *Purpureocillium*

**Table:** 

1	Species that grow on spiders and insect forming synnemata of colours purple to lilac	**2**
–	Species isolated, clinical specimens on human, animals, soil and crystals salt	**3**
2	Synnemata lilac coloured, conidia ellipsoid to cylindrical 4.8–5.6 × 1.6–2.4 μm; parasite trapdoor spiders; sexual state *Cordycepscylindrica*	***Purpureocilliumatypicola***
–	Synnemata lilac coloured, conidia ellipsoid 2.5–4 × 1.4–1.8 μm; parasite on cadavers of cicada adults; sexual state in *Cordycepsryogamimontana*	***Purpureocilliumtakamizusanense***
3	*Acremonium*-like synanamorph absent	**4**
–	*Acremonium*-like synanamorph present	**5**
4	Phialides 8–10 (14) × 2–3 μm, conidia globose, 2–2.5 μm	***Purpureocilliumroseum***
–	Phialides 6.8–11.7 × 2.6–4.0 μm, conidia ellipsoidal, 2.7–4.2 × 2.0–2.4 μm	***Purpureocilliumzongqii***
5	Conidia subglobose with apiculate base or limoniform	**6**
–	Conidia ellipsoidal to fusiform	***Purpureocilliumlilacinum***
6	Conidia 2–3 × 2–2.5 μm	***Purpureocilliumlavendulum***
–	Conidia 3.5–5.5 × 3–4.5 μm	***Purpureocilliumsodanum***

## ﻿Discussion

The genus *Perennicordyceps* was proposed to accommodate four species of *Polycephalomyces*, *Polycephalomycesprolificus* (Kobayasi) Kepler & Spatafora, *P.cuboideus* (Kobayasi & Shimizu) Kepler & Spatafora, *P.paracuboideus* (S. Ban, Sakane & Nakagiri) Kepler & Spatafora and *P.ryogamiensis* (Kobayasi & Shimizu) Kepler & Spatafora (Matočec et al. 2014). Species in the genus usually known as entomopathogenic fungi with host in the orders Coleoptera and Hemiptera (Matočec et al. 2014). Xiao et al. (2023) reported a new species, *Perennicordycepslutea* Y.B. Wang, H. Yu & Y.P. Xiao and a new combined species, *P.elaphomyceticola* (W.Y. Chuang, H.A. Ariyaw., J.I. Yang & Stadler) Y.P. Xiao & K.D. Hyde, which were both recorded as fungicolous. In the present study, the new species *P.zongqii* with Lepidoptera larvae was introduced. *Cordyceps*-like fungi have evolved adaptively through nutrient exchange and the ultimate goal is in the search of ideal food (Moonjely et al. 2016; Vidhate et al. 2023). Whether the new species has coevolved with its hosts and has special metabolizing processes is worthy of further research.

The genus *Purpureocillium* was established to accommodate *Paecilomyceslilacinus* Thom (Luangsa-ard et al. 2011). Members of *Purpureocillium* have a global distribution, especially for the type species *P.lilacinum*, which is commonly isolated from soil, decaying vegetation, insects, nematodes and laboratory air (as contaminant) (Luangsa-ard et al. 2011; Perdomo et al. 2013; Quandt et al. 2014; Ban et al. 2015b; Calvillo‐Medina et al. 2021). In the present study, the new species *P.zongqii* was isolated from soil in Monkey-Ear Tiankeng. Tiankeng acts as a refugium for biodiversity amid changing global climate and some ancient (*Alsophilaspinulosa* (Wall. ex Hook.) R. M. Tryon) and unique plants (cool-adapted plants) were present in Tiankeng (Shui et al. 2015; Bátori et al. 2017; Pu et al. 2019; Shen et al. 2020). Whether the new species is more ancient than others, has coevolved with its environment and has special metabolizing processes is worthy of further research.

The karst region in southwestern China is one of the 36 biodiversity hotspots in the world, nurturing a large number of endemic species (Delgado Baquerizo et al. 2020), especially in the special eco-environment, Tiankeng and the valley. The species that exist in these habitats often have very narrow distribution areas and very small populations, and urgently need to be protected (Wu and Zhang 2020). In the present study, species in the families Polycephalomycetaceae and Ophiocordycipitaceae were introduced in Tiankeng and the valley for the first time. Further attention needs to be paid to the diversity of other *Cordyceps*-like fungi in the special eco-environment of karst region.

## Supplementary Material

XML Treatment for Perennicordyceps zongqii

XML Treatment for Purpureocillium zongqii

## ﻿Additional information

### Author ORCIDs

Wan-Hao Chen https://orcid.org/0000-0001-7240-6841

Jian-Dong Liang https://orcid.org/0000-0002-3939-3900

Yan-Feng Han https://orcid.org/0000-0002-8646-3975

### Data availability

All of the data that support the findings of this study are available in the main text or Supplementary Information.

## Supplementary materials

Supplementary material 1 Alignment dataset 1 Data type fas https://binary.pensoft.net/file/1169644This dataset is made available under the Open Database License (http://opendatacommons.org/licenses/odbl/1.0/). The Open Database License (ODbL) is a license agreement intended to allow users to freely share, modify, and use this Dataset while maintaining this same freedom for others, provided that the original source and author(s) are credited.Wan-Hao Chen, Dan Li, Jian-Dong Liang, Xiu-Xiu Ren, Jie-Hong Zhao, Yan-Feng Han

Supplementary material 2 Alignment dataset 2 Data type fas https://binary.pensoft.net/file/1169645This dataset is made available under the Open Database License (http://opendatacommons.org/licenses/odbl/1.0/). The Open Database License (ODbL) is a license agreement intended to allow users to freely share, modify, and use this Dataset while maintaining this same freedom for others, provided that the original source and author(s) are credited.Wan-Hao Chen, Dan Li, Jian-Dong Liang, Xiu-Xiu Ren, Jie-Hong Zhao, Yan-Feng Han

Supplementary material 3 Alignment dataset 3 Data type fas https://binary.pensoft.net/file/1169646This dataset is made available under the Open Database License (http://opendatacommons.org/licenses/odbl/1.0/). The Open Database License (ODbL) is a license agreement intended to allow users to freely share, modify, and use this Dataset while maintaining this same freedom for others, provided that the original source and author(s) are credited.Wan-Hao Chen, Dan Li, Jian-Dong Liang, Xiu-Xiu Ren, Jie-Hong Zhao, Yan-Feng Han

Supplementary material 4 Alignment dataset 4 Data type fas https://binary.pensoft.net/file/1169647This dataset is made available under the Open Database License (http://opendatacommons.org/licenses/odbl/1.0/). The Open Database License (ODbL) is a license agreement intended to allow users to freely share, modify, and use this Dataset while maintaining this same freedom for others, provided that the original source and author(s) are credited.Wan-Hao Chen, Dan Li, Jian-Dong Liang, Xiu-Xiu Ren, Jie-Hong Zhao, Yan-Feng Han

Supplementary material 5 Alignment dataset 5 Data type nxs https://binary.pensoft.net/file/1169648This dataset is made available under the Open Database License (http://opendatacommons.org/licenses/odbl/1.0/). The Open Database License (ODbL) is a license agreement intended to allow users to freely share, modify, and use this Dataset while maintaining this same freedom for others, provided that the original source and author(s) are credited.Wan-Hao Chen, Dan Li, Jian-Dong Liang, Xiu-Xiu Ren, Jie-Hong Zhao, Yan-Feng Han

## References

[B1] Ban S Sakane T Toyama K Nakagiri A (2009) Teleomorph–anamorph relationships and reclassification of *Cordycepscuboidea* and its allied species. Mycoscience 50(4): 261–272. 10.1007/S10267-008-0480-Y

[B2] Ban S Sakane T Nakagiri A (2015a) Three new species of *Ophiocordyceps* and overview of anamorph types in the genus and the family Ophiocordyceptaceae. Mycological Progress 14(1): 1017. 10.1007/s11557-014-1017-8

[B3] Ban S Azuma Y Sato H Suzuki KI Nakagiri A (2015b) *Isariatakamizusanensis* is the anamorph of *Cordycepsryogamimontana*, warranting a new combination, *Purpureocilliumtakamizusanense* comb. nov. International Journal of Systematic and Evolutionary Microbiology 65(8): 2459–2465. 10.1099/ijs.0.000284 25911534

[B4] Bátori Z Vojtkó A Farkas T Szabó A Havadtői K Vojtkó AE Tölgyesi C Cseh V Erdős L Maák IE Keppel G (2017) Large-and small-scale environmental factors drive distributions of cool-adapted plants in karstic microrefugia. Annals of Botany 119(2): 301–309. 10.1093/aob/mcw233 28025290 PMC5321062

[B5] Calvillo‐Medina RP Ponce‐Angulo DG Raymundo T Müller‐Morales CA Escudero‐Leyva E Campos Guillén J Bautista‐de Lucio VM (2021) *Purpureocilliumroseum* sp. nov. A new ocular pathogen for humans and mice resistant to antifungals. Mycoses 64(2): 162–173. 10.1111/myc.13198 33064905

[B6] Castlebury LA Rossman AY Sung GH Hyten AS Spatafora JW (2004) Multigene phylogeny reveals new lineage for *Stachybotryschartarum*, the indoor air fungus. Mycological Research 108(8): 864–872. 10.1017/S0953756204000607 15449591

[B7] Chen WH Liu C Han YF Liang JD Tian WY Liang ZQ (2019) Three novel insect-associated species of *Simplicillium* (Cordycipitaceae, Hypocreales) from Southwest China. MycoKeys 58: 83–102. 10.3897/mycokeys.58.37176 31592222 PMC6775174

[B8] Chen WH Han YF Liang JD Liang ZQ (2021a) Taxonomic and phylogenetic characterizations reveal four new species of *Simplicillium* (Cordycipitaceae, Hypocreales) from Guizhou, China. Scientific Reports 11(1): 1–12. 10.1038/s41598-021-94893-z 34316013 PMC8316311

[B9] Chen WH Han YF Liang JD Tian WY Liang ZQ (2021b) Multi-gene phylogenetic evidence indicates that *Pleurodesmospora* belongs in Cordycipitaceae (Hypocreales, Hypocreomycetidae) and *Pleurodesmosporalepidopterorum* sp. nov. on pupa from China. MycoKeys 80: 45–55. 10.3897/mycokeys.80.66794 34035655 PMC8124063

[B10] Chen WH Liang JD Ren XX Zhao JH Han YF Liang ZQ (2021c) Cryptic diversity of *Isaria*-like species in Guizhou, China. Life (Basel, Switzerland) 11(10): 1093. 10.3390/life11101093 34685462 PMC8539930

[B11] Chen WH Liang JD Han YF Zou X Zhang YJ Liang ZQ (2021d) Research overviews of *Cordyceps*: Past, present and future. Junwu Xuebao 40(11): 2894–2905.

[B12] Chen WH Liang JD Ren XX Zhao JH Han YF Liang ZQ (2022a) Phylogenetic, ecological and morphological characteristics reveal two new spider-associated genera in Clavicipitaceae. MycoKeys 91: 49–66. 10.3897/mycokeys.91.86812 36760893 PMC9849053

[B13] Chen WH Liang JD Ren XX Zhao JH Han YF Liang ZQ (2022b) Species diversity of *Cordyceps*-like fungi in the Tiankeng karst region of China. Microbiology Spectrum 10(5): e01975–e22. 10.1128/spectrum.01975-22 PMC9603550 36094103

[B14] Chen WH Liang JD Ren XX Zhao JH Han YF Liang ZQ (2022c) Multigene phylogeny, phylogenetic network, and morphological characterizations reveal four new arthropod-associated *Simplicillium* species and their evolutional relationship. Frontiers in Microbiology 13: 950773. 10.3389/fmicb.2022.950773 PMC9578668 36267186

[B15] Chen WH Liang JD Ren XX Zhao JH Han YF (2023) Two new species of *Samsoniella* (Cordycipitaceae, Hypocreales) from the Mayao River Valley, Guizhou, China. MycoKeys 99: 209–226. 10.3897/mycokeys.99.109961 37744955 PMC10517413

[B16] Chen W Tang Y Liu T Hu H Ou C Hu Q Weng Q (2024) *Purpureocilliumjiangxiense* sp. nov.: Entomopathogenic effects on *Ostriniafurnacalis* and *Galleriamellonella*. Microorganisms 12(6): 1041. 10.3390/microorganisms12061041 PMC11205419 38930423

[B17] Crous PW Gams W Stalpers JA Robert V Stegehuis G (2004) MycoBank: An online initiative to launch mycology into the 21^st^ century. Studies in Mycology 50(1): 19–22.

[B18] Dai YD Chen SQ Wang YB Wang Y Yang ZL Yu H (2024) Molecular phylogenetics of the *Ophiocordycepssinensis*-species complex lineage (Ascomycota, Hypocreales), with the discovery of new species and predictions of species distribution. IMA Fungus 15(1): 2. 10.1186/s43008-023-00131-8 38336758 PMC10858606

[B19] Delgado-Baquerizo M Reich PB Trivedi C Eldridge DJ Abades S Alfaro DF Bastida F Berhe AA Cutler NA Gallardo A García-Velázquez L Hart SC Hayes PE He JZ Hseu ZY Hu HW Kirchmair M Neuhauser S Pérez CA Reed SC Santos F Sullivan BW Trivedi P Wang JT Weber-Grullon L Williams MA Singh BK (2020) Multiple elements of soil biodiversity drive ecosystem functions across biomes. Nature Ecology & Evolution 4(2): 210–220. 10.1038/s41559-019-1084-y 32015427

[B20] Dong QY Wang Y Wang ZQ Tang DX Zhao ZY Wu HJ Yu H (2022) Morphology and phylogeny reveal five novel species in the genus *Cordyceps* (Cordycipitaceae, Hypocreales) From Yunnan, China. Frontiers in Microbiology 13: 846909. 10.3389/fmicb.2022.846909 PMC9044072 35495705

[B21] Drummond A Rambaut A (2007) BEAST: Bayesian evolutionary analysis by sampling trees. BMC Evolutionary Biology 7(1): e214. 10.1186/1471-2148-7-214 PMC2247476 17996036

[B22] Hyde KD Hongsanan S Jeewon R Bhat DJ McKenzie EHC Jones EBG Phookamsak R Ariyawansa HA Boonmee S Zhao Q Abdel-Aziz FA Abdel-Wahab MA Banmai S Chomnunti P Cui BK Daranagama DA Das K Dayarathne MC de Silva NI Dissanayake AJ Doilom M Ekanayaka AH Gibertoni TB Góes-Neto A Huang SK Jayasiri SC Jayawardena RS Konta S Lee HB Li WJ Lin CG Liu JK Lu YZ Luo ZL Manawasinghe IS Manimohan P Mapook A Niskanen T Norphanphoun C Papizadeh M Perera RH Phukhamsakda C (2016) Fungal diversity notes 367–490: Taxonomic and phylogenetic contributions to fungal taxa. Fungal Diversity 80: 1–270. 10.1007/s13225-016-0373-x

[B23] Kalyaanamoorthy S Minh BQ Wong TK Von Haeseler A Jermiin LS (2017) ModelFinder: Fast model selection for accurate phylogenetic estimates. Nature Methods 14(6): 587–589. 10.1038/nmeth.4285 28481363 PMC5453245

[B24] Katoh K Standley DM (2013) MAFFT multiple sequence alignment software version 7: Improvements in performance and usability. Molecular Biology and Evolution 30(4): 772–780. 10.1093/molbev/mst010 23329690 PMC3603318

[B25] Kepler R Ban S Nakagiri A Bischoff J Hywel-Jones N Owensby CA Spatafora JW (2013) The phylogenetic placement of hypocrealean insect pathogens in the genus *Polycephalomyces*: An application of One Fungus One Name. Fungal Biology 117(9): 611–622. 10.1016/j.funbio.2013.06.002 24012301

[B26] Li Y Song B Wang K Deng CY Wang YH Yuan Y Wen HS Li TH (2021) Current status and protection of threatened *Cordyceps* s. l. species in China. Acta Edulis Fungi 28(2): 113–122.

[B27] Li CH Yang SS Deng WQ Lin QY (2023) *Cordyceps* sensu lato and their domestication and cultivation in China. Acta Edulis Fungi 30(5): 113–148.

[B28] Liang JD Han YF Zhang JW Du W Liang ZQ Li ZZ (2011) Optimal culture conditions for keratinase production by a novel thermophilic *Myceliophthorathermophila* strain GZUIFR-H49-1. Journal of Applied Microbiology 110(4): 871–880. 10.1111/j.1365-2672.2011.04949.x 21241422

[B29] Liu YJ Whelen S Hall BD (1999) Phylogenetic relationships among ascomycetes: Evidence from an RNA polymerase II subunit. Molecular Biology and Evolution 16(12): 1799–1808. 10.1093/oxfordjournals.molbev.a026092 10605121

[B30] Luangsa-ard J Houbraken J van Doorn T Hong SB Borman AM Hywel-Jones NL Samson RA (2011) *Purpureocillium*, a new genus for the medically important *Paecilomyceslilacinus*. FEMS Microbiology Letters 321(2): 141–149. 10.1111/j.1574-6968.2011.02322.x 21631575

[B31] Matočec N Kušan I Ozimec R (2014) The genus *Polycephalomyces* (Hypocreales) in the frame of monitoring Veternica cave (Croatia) with a new segregate genus *Perennicordyceps*. Ascomycete. Org : Revue Internationale pour la Taxinomie des Ascomycota 6: 125–133.

[B32] Ming DQ Luo LY He XX Wang MS Fang WX Chen SF Chen WH Han YF Liang ZQ (2021) *Paracremoniumlepidopterorum*, a new insect-associated fungus. Phytotaxa 524(2): 85–91. 10.11646/phytotaxa.524.2.2

[B33] Moonjely S Barelli L Bidochka MJ (2016) Insect pathogenic fungi as endophytes. Advances in Genetics 94: 107–135. 10.1016/bs.adgen.2015.12.004 27131324

[B34] Özkan K Gulsoy S Mert A Ozturk M Muys B (2010) Plant distribution-altitude and landform relationships in karstic sinkholes of Mediterranean region of Turkey. Journal of Environmental Biology 31(1): 51–60. 20648813

[B35] Peng XC Xiao YP Zhang Y Chomnunti P Tangtrakulwanich K Wen TC (2023) *Cordycepspoluscapitis* sp. nov., an ant-pathogenic fungus from Guizhou, China. Phytotaxa 599(4): 239–251. 10.11646/phytotaxa.599.4.3

[B36] Peng XC Wen TC Wei DP Liao YH Wang Y Zhang X Wang GY Zhou Y Tangtrakulwanich K Liang JD (2024) Two new species and one new combination of *Ophiocordyceps* (Hypocreales, Ophiocordycipitaceae) in Guizhou. MycoKeys 102: 245–266. 10.3897/mycokeys.102.113351 38463694 PMC10921062

[B37] Perdomo H Cano J Gené J García D Hernández M Guarro J (2013) Polyphasic analysis of *Purpureocilliumlilacinum* isolates from different origins and proposal of the new species *Purpureocilliumlavendulum*. Mycologia 105(1): 151–161. 10.3852/11-190 22893638

[B38] Pu GZ Lv YN Dong L Zhou LW Huang KC Zeng DJ Mo L Xu GP (2019) Profiling the bacterial diversity in a typical karst Tiankeng of China. Biomolecules 9(5): 187. 10.3390/biom9050187 31091762 PMC6572312

[B39] Qu JJ Zou X Cao W Xu ZS Liang ZQ (2021) Two new species of *Hirsutella* (Ophiocordycipitaceae, Sordariomycetes) that are parasitic on lepidopteran insects from China. MycoKeys 82: 81–96. 10.3897/mycokeys.82.66927 34408539 PMC8367965

[B40] Quandt CA Kepler RM Gams W Araújo JP Ban S Evans HC Hughes D Humber R Hywel-Jones N Li ZZ Luangsa-ard JJ Rehner SA Sanjuan T Sato H Shrestha B Sung GH Yao YJ Zare R Spatafora JW (2014) Phylogenetic-based nomenclatural proposals for Ophiocordycipitaceae (Hypocreales) with new combinations in *Tolypocladium*. IMA Fungus 5(1): 121–134. 10.5598/imafungus.2014.05.01.12 PMC4107890 25083412

[B41] Quesada-Moraga E Navas-Cortés JA Maranhao EA Ortiz-Urquiza A Santiago-Álvarez C (2007) Factors affecting the occurrence and distribution of entomopathogenic fungi in natural and cultivated soils. Mycological Research 111(8): 947–966. 10.1016/j.mycres.2007.06.006 17766099

[B42] Ronquist F Teslenko M van der Mark P Ayres DL Darling A Höhna S Larget B Liu L Suchard MA Huelsenbeck JP (2012) MrBayes 3.2: Efficient Bayesian phylogenetic inference and model choice across a large model space. Systematic Biology 61(3): 539–542. 10.1093/sysbio/sys029 22357727 PMC3329765

[B43] Schoch CL Seifert KA Huhndorf S Robert V Spouge JL Levesque CA Chen W Bolchacova E Voigt K Crous PW Miller AN Wingfield MJ Aime MC An K-D Bai F-Y Barreto RW Begerow D Bergeron M-J Blackwell M Boekhout T Bogale M Boonyuen N Burgaz AR Buyck B Cai L Cai Q Cardinali G Chaverri P Coppins BJ Crespo A Cubas P Cummings C Damm U de Beer ZW de Hoog GS Del-Prado R Dentinger B Diéguez-Uribeondo J Divakar PK Douglas B Dueñas M Duong TA Eberhardt U Edwards JE Elshahed MS Fliegerova K Furtado M García MA Ge Z-W Griffith GW Griffiths K Groenewald JZ Groenewald M Grube M Gryzenhout M Guo L-D Hagen F Hambleton S Hamelin RC Hansen K Harrold P Heller G Herrera C Hirayama K Hirooka Y Ho H-M Hoffmann K Hofstetter V Högnabba F Hollingsworth PM Hong S-B Hosaka K Houbraken J Hughes K Huhtinen S Hyde KD James T Johnson EM Johnson JE Johnston PR Jones EBG Kelly LJ Kirk PM Knapp DG Kõljalg U Kovács GM Kurtzman CP Landvik S Leavitt SD Liggenstoffer AS Liimatainen K Lombard L Luangsa-ard JJ Lumbsch HT Maganti H Maharachchikumbura SSN Martin MP May TW McTaggart AR Methven AS Meyer W Moncalvo J-M Mongkolsamrit S Nagy LG Nilsson RH Niskanen T Nyilasi I Okada G Okane I Olariaga I Otte J Papp T Park D Petkovits T Pino-Bodas R Quaedvlieg W Raja HA Redecker D Rintoul TL Ruibal C Sarmiento-Ramírez JM Schmitt I Schüßler A Shearer C Sotome K Stefani FOP Stenroos S Stielow B Stockinger H Suetrong S Suh S-O Sung G-H Suzuki M Tanaka K Tedersoo L Telleria MT Tretter E Untereiner WA Urbina H Vágvölgyi C Vialle A Vu TD Walther G Wang Q-M Wang Y Weir BS Weiß M White MM Xu J Yahr R Yang ZL Yurkov A Zamora J-C Zhang N Zhuang W-Y Schindel D (2012) Nuclear ribosomal internal transcribed spacer (ITS) region as a universal DNA barcode marker for Fungi. Proceedings of the National Academy of Sciences of the United States of America 109(16): 6241–6246. 10.1073/pnas.1117018109 22454494 PMC3341068

[B44] Shen LN Hou MF Xu WB Huang YF Liang SC Zhang YH Jiang ZC Chen WH (2020) Research on flora of seed plants in Dashiwei Karst Tiankeng group of Leye. Guangxi Guihaia 40: 751–764.

[B45] Shui W Chen YP Wang YW Su ZA Zhang S (2015) Origination, study progress and prospect of karst tiankeng research in China. Acta Geographica Sinica 70: 431–446.

[B46] Song B Wu XL Li TH (2011) Species of *Cordyceps*-like fungi from Guangxi, China. Guizhou Science 29(1): 47–51.

[B47] Su Y Tang Q Mo F Xue Y (2017) Karst tiankengs as refugia for indigenous tree flora amidst a degraded landscape in southwestern China. Scientific Reports 7(1): 1–10. 10.1038/s41598-017-04592-x 28652612 PMC5484678

[B48] Sun JZ Liu XZ McKenzie EH Jeewon R Liu JK Zhang XL Zhao Q Hyde KD (2019) Fungicolous fungi: Terminology, diversity, distribution, evolution, and species checklist. Fungal Diversity 95(1): 1–94. 10.1007/s13225-019-00422-9

[B49] Tamura K Stecher G Peterson D Filipski A Kumar S (2013) MEGA6: Molecular evolutionary genetics analysis version 6.0. Molecular Biology and Evolution 30(12): 2725–2729. 10.1093/molbev/mst197 24132122 PMC3840312

[B50] Tang D Xu Z Wang Y Wang Y Tran NL Yu H (2023a) Multigene phylogeny and morphology reveal two novel zombie-ant fungi in *Ophiocordyceps* (Ophiocordycipitaceae, Hypocreales). Mycological Progress 22(4): 22. 10.1007/s11557-023-01874-9

[B51] Tang D Zhao J Lu Y Wang Z Sun T Liu Z Yu H (2023b) Morphology, phylogeny and host specificity of two new *Ophiocordyceps* species belonging to the “zombie-ant fungi” clade (Ophiocordycipitaceae, Hypocreales). MycoKeys 99: 269–296. 10.3897/mycokeys.99.107565 37881189 PMC10594121

[B52] Tang D Huang O Zou W Wang Y Wang Y Dong Q Sun T Yang G Yu H (2023c) Six new species of zombie-ant fungi from Yunnan in China. IMA Fungus 14(1): 9. 10.1186/s43008-023-00114-9 37170179 PMC10173673

[B53] Trifinopoulos J Nguyen LT von Haeseler A Minh BQ (2016) W-IQ-TREE: A fast online phylogenetic tool for maximum likelihood analysis. Nucleic Acids Research 44(W1): W232–W235. 10.1093/nar/gkw256 PMC4987875 27084950

[B54] Vaidya G Lohman DJ Meier R (2011) SequenceMatrix: Concatenation software for the fast assembly of multi-gene datasets with character set and codon information. Cladistics 27(2): 171–180. 10.1111/j.1096-0031.2010.00329.x 34875773

[B55] Vidhate RP Dawkar VV Punekar SA Giri AP (2023) Genomic determinants of entomopathogenic fungi and their involvement in pathogenesis. Microbial Ecology 85(1): 49–60. 10.1007/s00248-021-01936-z 34977966

[B56] Vilgalys R Hester M (1990) Rapid genetic identification and mapping of enzymatically amplified ribosomal DNA from several *Cryptococcus* species. Journal of Bacteriology 172(8): 4238–4246. 10.1128/jb.172.8.4238-4246.1990 2376561 PMC213247

[B57] Wang YH Sayaka B Wang WJ Li Y Wang K Kirk PM Bushley KE Dong CH Hawksworth DL Yao YJ (2021) *Pleurocordyceps* gen. nov. for a clade of fungi previously included in *Polycephalomyces* based on molecular phylogeny and morphology. Journal of Systematics and Evolution 59(5): 1065–1080. 10.1111/jse.12705

[B58] Wang Z Wang Y Dong Q Fan Q Dao VM Yu H (2022) Morphological and phylogenetic characterization reveals five new species of *Samsoniella* (Cordycipitaceae, Hypocreales). Journal of Fungi (Basel, Switzerland) 8(7): 747. 10.3390/jof8070747 35887502 PMC9321185

[B59] Wang Y Wang ZQ Luo R Souvanhnachit S Thanarut C Dao VM Yu H (2024a) Species diversity and major host/substrate associations of the genus *Akanthomyces* (Hypocreales, Cordycipitaceae). MycoKeys 101: 113–141. 10.3897/mycokeys.101.109751 38269036 PMC10806914

[B60] Wang HY Li X Dong CB Zhang YW Chen WH Liang JD Han YF (2024b) Two new species of Sordariomycetes (Chaetomiaceae and Nectriaceae) from China. MycoKeys 102: 301–315. 10.3897/mycokeys.102.114480 38495535 PMC10940860

[B61] White TJ Bruns T Lee S Taylor J (1990) Amplification and direct sequencing of fungal ribosomal RNA genes for phylogenetics. In: Innis MA Gelfand DH Sninsky JJ White TJ (Eds) PCR protocols: a guide to methods and applications.Academic Press, New York, 315–322. 10.1016/B978-0-12-372180-8.50042-1

[B62] Wu J Zhang CH (2020) Research status and prospect of karst Tiankengs in Guizhou Province. Carsologica Sinica 39(1): 119–126.

[B63] Xiao YP Hongsanan S Hyde KD Brooks S Xie N Long FY Wen TC (2019) Two new entomopathogenic species of *Ophiocordyceps* in Thailand. MycoKeys 47: 53–74. 10.3897/mycokeys.47.29898 PMC6395454 30828254

[B64] Xiao YP Wang YB Hyde KD Eleni G Sun JZ Yang Y Meng J Yu H Wen TC (2023) Polycephalomycetaceae, a new family of clavicipitoid fungi segregates from Ophiocordycipitaceae. Fungal Diversity 120(1): 1–76. 10.1007/s13225-023-00517-4

[B65] Xiao YP Yang Y Jayawardena RS Gentekaki E Peng XC Luo ZL Lu YZ (2024) Four novel *Pleurocordyceps* (Polycephalomycetaceae) species from China. Frontiers in Microbiology 14: 1256967. 10.3389/fmicb.2023.1256967 PMC10807425 38268701

[B66] Zhang D Gao F Jakovlic I Zou H Zhang J Li WX Wang GT (2020) PhyloSuite: An integrated and scalable desktop platform for streamlined molecular sequence data management and evolutionary phylogenetics studies. Molecular Ecology Resources 20(1): 348–355. 10.1111/1755-0998.13096 31599058

[B67] Zhang Y Wen T Xiao Y Yang Y Peng X (2023) A new species of *Papiliomyces* (Clavicipiteae, Hypocreales) from China. Biodiversity Data Journal 11: e86868. 10.3897/BDJ.11.e86868 PMC10265218 37325231

[B68] Zhou YM Zhi JR Qu JJ Zou X (2022) Estimated divergence times of *Lecanicillium* in the family Cordycipitaceae provide insights into the attribution of *Lecanicillium*. Frontiers in Microbiology 13: 859886. 10.3389/fmicb.2022.859886 PMC9121009 35602068

[B69] Zhu GS Liu ZY Wu XL Liu YX (2004) The research of *Cordyceps* in the karst area of Yunnan, Guizhou and Guangxi Provinces in China. Guizhou Science 1: 27–30.

[B70] Zou X Liu AY Liang ZQ Han YF Yang M (2010) *Hirsutellaliboensis*, a new entomopathogenic species affecting Cossidae (Lepidoptera) in China. Mycotaxon 111(1): 39–44. 10.5248/111.39

[B71] Zou W Tang D Xu Z Huang O Wang Y Tran NL Yu H (2022) Multigene phylogeny and morphology reveal *Ophiocordycepshydrangea* sp. nov. and *Ophiocordycepsbidoupensis* sp. nov. (Ophiocordycipitaceae). MycoKeys 92: 109–130. 10.3897/mycokeys.92.86160 36761313 PMC9849067

